# The Advocacy-Inquiry Rubric (AIR): a standard to build debriefing and feedback skills

**DOI:** 10.1186/s41077-025-00381-z

**Published:** 2025-11-24

**Authors:** Clément Buléon, Demian Szyld, Robert Simon, Lon Setnik, Walter J. Eppich, Mary Fey, James A. Lipshaw, Janice C. Palaganas, Jenny W. Rudolph, Advocacy Inquiry Interest Group

**Affiliations:** 1 Center for Medical Simulation, Boston, MA USA; 2 Center for Medical Simulation of Liege, University of Liege, Liège, Belgium; 3 Emergency Department, University Hospital of Liege, Liege, Belgium; 4 Department of Emergency Medicine, Boston University Chobanian and Avedisian School of Medicine, Boston, MA USA; 5 Faculty of Medicine, Dentistry and Health Sciences, University of Melbourne, Melbourne, Australia; 6 Department of Anesthesia, Critical Care and Pain Medicine, Beth Israel Deaconess Hospital, Boston, MA USA; 7 Massachusetts General Hospital Institute for Health Professions, Boston, MA USA; 8 Harvard Medical School, Boston, MA USA

**Keywords:** Delphi method, Debriefing, Feedback, Assessment, Simulation, Advocacy inquiry, Rubric, Rater training

## Abstract

**Background:**

Teaching and learning debriefing and feedback skills—especially to a level of mastery—is challenging without an agreed-upon standard. There are a number of rating scales and rubrics to identify and evaluate debriefing and feedback skills that focus on an entire feedback or debriefing conversation. However, there is no rubric to assess and provide feedback on one of these conversations' most widely used microskills, the Advocacy-Inquiry technique. This study aimed to develop and preliminarily test the Advocacy-Inquiry Rubric (AIR)—a tool designed to support the teaching, coaching, and assessment of Advocacy-Inquiry, a widely used yet challenging debriefing microskill—through an international expert consensus process.

**Method:**

Using a four-round Delphi process, we achieved expert consensus on the behavioral markers of effective and ineffective Advocacy-Inquiry techniques. Thirty-nine experts from 13 countries identified and refined a set of key behavioral anchors for each of Advocacy-Inquiry’s five elements: Preview, Observation, Point of View, Inquiry, and Listen. These descriptors were embedded first in a seven-point numeric Behaviorally Anchored Rating Scale, then in a three-point emoji-based version, and finally in a teaching and learning version. The AIR underwent two rounds of usability testing and inter-rater testing of the emoji version. Using an interpretation-use argument approach, evidence was collected for AIR’s validity across scoring, generalization, extrapolation, and implication.

**Results:**

The Delphi process established descriptors for each element of Advocacy-Inquiry, categorized by proficiency level (beginner to advanced). Usability testing enhanced the AIR’s graphic layout to support both numeric ratings and formative feedback. The AIR was adapted into three tailored versions: a numeric AIR for detailed evaluation and progress tracking, an emoji AIR for peer assessment, and a teaching and learning AIR. Evidence for validity was assessed, highlighting both strengths and gaps.

**Conclusion:**

AIR is an empirical rubric based on expert-derived criteria to support teaching, coaching, and assessing Advocacy-Inquiry microskills. The AIR offers a structured framework for self-, peer-, and mentor-led feedback and assessment to enhance a core skill of facilitators. By anchoring assessments in clear behavioral descriptors, the AIR aims to improve the quality of feedback and debriefing conversations. Future work should focus on rater training, reliability testing, and exploring the AIR’s impact on real-world outcomes.

**Supplementary Information:**

The online version contains supplementary material available at 10.1186/s41077-025-00381-z.

## Introduction

Teaching and learning debriefing skills are challenging without an agreed-upon standard. A number of rating scales and rubrics help identify and evaluate entire feedback or debriefing conversations [1–5]. Although helpful, these tools lack the granularity needed to develop expertise in a widely used set of microskills, the Advocacy-Inquiry technique. Without a rubric that focuses on these microskills, the fine-grained feedback and deliberate practice needed to master them remain elusive.

For individuals who lead feedback and debriefing conversations as well as simulation program leaders, a trustworthy, easy-to-use rubric could help develop, standardize, and assess faculty’s debriefing and feedback skills. Developing an explicit and transparent shared mental model of the standard serves the goals of teaching, learning, and assessment. Such a rubric directs learners’, teachers’, and assessors' attentions and aids them in sampling behavior, categorizing it, and assessing it [6]. Although Advocacy-Inquiry is a widely taught and applied microskill in simulation-based education, its nuanced, multi-step structure and reliance on both relational and cognitive skills make it challenging to master and assess without a dedicated, behaviorally anchored rubric.

The purpose of this paper is to describe the goals and the development process of a tool called the Advocacy-Inquiry Rubric (AIR). The main goal of the AIR is to facilitate the teaching and learning of formative and summative assessment of Advocacy-Inquiry skills. The process we describe includes developing behavioral descriptors using a Delphi method, guided by the goal of transparency in building an argument for the validity of the AIR, and preliminary testing of its application in a peer feedback context.

### The purpose and use of the Advocacy-Inquiry technique

Advocacy-Inquiry, first introduced into healthcare simulation debriefing in 2006 [7], is now a foundational skill in many debriefing frameworks and simulation facilitator training programs [8–12]. It is particularly effective in fostering reflective practice, encouraging critical thinking, and bridging perspectives in feedback conversations and debriefings [13, 14]. It provides opportunities for both the conversation facilitator and partners to share their assumptions, goals, and values, an integral part of effective dialogic conversations.

For the purposes of this paper, we use these terms: Advocacy-Inquiry refers to the conversational technique, and Advocacy-Inquiry Rubric (AIR) refers to the rubric. We also use the term “facilitator” to refer to the person instructing, coaching, facilitating, or debriefing [15]. Research highlights the value of facilitators’ transparently sharing their goals and inviting input, which can help manage the conversation partner’s mental effort (cognitive load) and encourage them to share their thoughts and goals openly [16–18]. Advocacy-Inquiry can drive transformative conversations that facilitate the bridging of diverse perspectives [19].

The five-part Advocacy-Inquiry technique—Preview, Advocacy (observation and point of view), Inquiry, and Listen (Table 1)—builds on foundational work by Argyris [13], Torbert and Taylor [20], and our team’s teaching experience [7]. Previewing refines Torbert’s “framing” by clearly signaling goals, enhancing mutuality, and reducing cognitive load and uncertainty [21–23]. Advocacy promotes transparency [24] by sharing a specific observation and its implications, avoiding “guess what I’m thinking” questions. Inquiry and Listen foster curiosity, reflection, and connection [5].

**Table 1:** Description of the Advocacy-Inquiry Elements

Element of the Advocacy-Inquiry	Description of the elements’ objectives
Preview	Orients the conversation partner to the topic or focus of the conversation
Observation	Shares a specific, concrete description of behavior the facilitator has seen or heard
Point of view	Reveals the facilitator’s perspective on the consequences of an action
Inquiry	Explores the driver or “why” for the observed behavior, asking an open-ended question to elicit the conversation partner’s perspective
Listen	Acknowledges the conversation partner’s perspective; explores the reasoning behind the observed behavior

The purpose of this study was to develop the AIR by (1) defining the construct and intended uses, (2) achieving expert consensus on behavioral descriptors through a Delphi process, (3) designing three rubric versions (numeric, teaching and learning, emoji) to support formative and summative applications, and (4) conducting preliminary usability and reliability testing.

## Methods

This study is reported according to the ACCORD guidelines [25].

### AIR development

We sought to create a rubric to teach and learn Advocacy-Inquiry that would facilitate and strengthen “rater cognition,” a multifaceted process that includes selecting, categorizing, and rating behaviors [6]. Thus, the rubric would support not only assessment but also scaffold teaching activities. Drawing on constructivist models of adult learning, we include within our definition of “rater cognition” how individuals create and apply mental models of the skills in development. With this goal in mind, we developed the AIR starting with the five theoretically derived elements described above: preview, observation, point of view, inquiry, and listen. Our goal was to arrive at an expert consensus on the behavioral markers of an effective (or ineffective) Advocacy-Inquiry.

To design the rubric, we drew on the authors' team’s insights from training more than 1000 simulation educators to rate debriefings using the Debriefing Assessment for Simulation in Healthcare (DASH) [2] and from supervising its translation into eight languages. Trained raters “see through the eyes” of a rubric, a learned cognitive process that eases selecting and categorizing observations. Rater cognition and valid assessment both rely on cognitive processes that transition from observing a performance to creating narrative descriptions of behavior, then progressing to scoring, and finally to making evaluative judgments about readiness, competence, or mastery. This process benefits from tools that help assessors see and process information consistently, a key strength of Behaviorally Anchored Rating Scales (BARS). Assessors use predefined behavioral descriptors to align observations with the rubric’s criteria, thereby focusing attention on agreed behavioral descriptors in a way that enhances the consistency of rating [26].

We invited individuals in the Delphi group to nominate behavioral descriptors for each AIR element. We asked them to provide good and poor performance descriptors, guiding raters in translating observations into scores. This method aligns with criterion-referenced testing, focusing on defined behavioral domains rather than comparisons between examinees. We elicited and evaluated behavioral markers for each element using a multi-step process adapted from other health professions’ rubric creation processes [27, 28] (Table 2).

**Table 2:** Instrument Development Steps

Development Step	Activity
1. Identify an initial checklist or rubric	The five main elements of Advocacy-Inquiry: Preview, Observation, Point of View, Inquiry, and Listen
2. Clarify the construct and uses	The construct to be measured was *Advocacy-Inquiry Skill*, and the uses were to *provide formative and summative assessments* of facilitators-in-training to enhance debriefing and feedback conversations
3. Gather experts’ input and refine items through iterative rounds	Asked experts to propose behavioral anchors for each element, including qualitative descriptors for good and poor performance of each element. The *four-round Delphi* process iteratively refined descriptors, incorporating quantitative rankings and weighted scoring to finalize descriptors. Used the fourth Delphi round to finalize descriptors, including their assignment to beginner, intermediate, or advanced levels. (See Fig. 3 for details.)
4. Establish scoring systems	Establish an initial *seven-point Behaviorally Anchored Rating Scale (BARS) AIR* and a*teaching and learning AIR*, both with descriptors of effectiveness for good and poor performance for each rubric element
5. Pilot test instrument (usability)	Iteratively tested the usability and functionality of the AIR layout and scale with two volunteer cohorts in a facilitator training course
6. Test Emoji Scale	Tested the emoji scale’s effectiveness in scoring through a rater training and inter-rater agreement assessment process

*AIR* Advocacy-Inquiry Rubric

### Clarifying construct and interpretations built into the AIR

We developed the AIR through a Delphi study, usability testing within two cohorts of facilitators-in-training, and testing of the emoji version of the rubric [29]. We prioritized testing the emoji version of the rating scale because this was the format that users in our user testing found most comfortable for peer feedback. Simulation programs we work with have been clamoring for an easy-to-use peer-to-peer faculty development process. Using the “interpretation-use argument” approach [30], we provide validity evidence for the AIR across observation, scoring, generalization, extrapolation, and implication.

As a construct, the rubric aims to build Advocacy-Inquiry skills, defined as facilitators’ ability to reveal their thinking and to elicit and acknowledge learners’ thinking while maintaining psychological safety. The rubric seeks to guide interpretations as to whether facilitators demonstrate sufficient skills to achieve these goals.

For the purpose of the development, our goal was to clarify the intended uses of the AIR. There are two ways that it can be used: formatively and summatively. The AIR is designed to be a structured tool for guiding both skill development (formative) and competency evaluation in feedback and debriefing (summative).

#### Formative use

The AIR supports self-, peer-, and mentor-led assessments for skill improvement. Using qualitative descriptors or numeric benchmarks, users of the rubric compare recorded or live feedback conversations to AIR descriptors to identify strengths and gaps, and provide actionable feedback. This applies to facilitator training, self-assessment, or guiding faculty development programs.

#### Summative use

Numeric assessments determine whether facilitators-in-training demonstrate Advocacy-Inquiry skills required for entrustment decisions. This includes assessing progress or deciding if a facilitator may lead specific learning conversations.

### Delphi approach

**Fig. 1:**
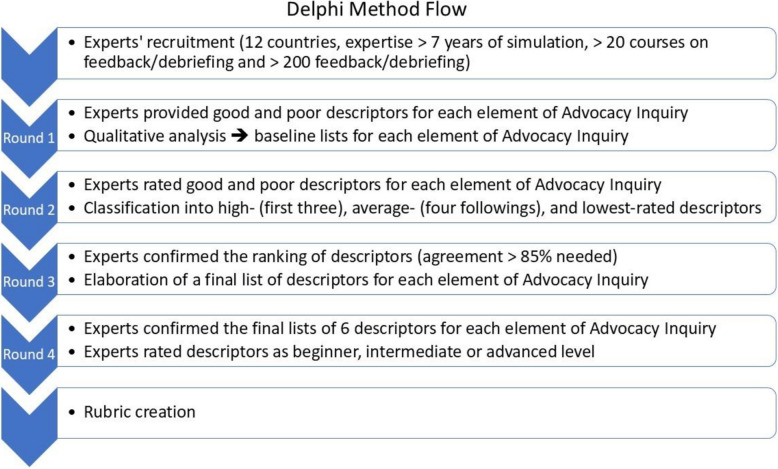
Delphi method flow

The behavioral descriptors for each element of the Advocacy-Inquiry technique were developed through a Delphi method (Fig. 1). We conducted the Delphi process in four rounds over 10 weeks, which included four key features: expert insider facilitation, response anonymity, qualitative and statistical analysis of expert responses, and sharing these analyses back to the experts at each round [29]. The project was submitted for institutional review and ethics oversight to the *Comité d’Ethique de la Recherche en Anesthésie-Réanimation* (CERAR, France Reference IRB 00010254–2019-063).

Investigators CB, JWR, and RS designed and conducted the Delphi study. In the Delphi method, researchers elicit expert perspectives on a focus of interest in multiple rounds [31, 32]. After each round, the investigators facilitating the study analyzed the experts' input from the previous round and explained the analysis process. Through these iterations, experts could revise earlier answers based on anonymized responses from other expert group members. The goal was to reach a consensus or convergence around key terms or descriptors of interest [32].

The study employed an "insider–outsider" research approach [33–35] leveraging the Delphi facilitators’ (CB, JWR, RS) expert knowledge of Advocacy-Inquiry. Investigators (CB, JWR, RS) and authors (MKF, DS, JCP, WE) were also participants in the Delphi process. This design capitalized on “inquiry from the inside” [36] by drawing on researchers’ pre-understanding from teaching and learning the technique [37]. Simultaneously, it incorporated “inquiry from the outside,” using qualitative and statistical methods to analyze anonymous data. Investigators’ (CB, JWR, RS) and authors’ (MKF, DS, JCP, WE) participation in the 4 rounds of the study was: CB (4), JWR (4), RS (2), MKF (4), DS (4), JCP (4), WE (3) combining immersion and global vision for robust findings.

### Expert panel selection

Experts were recruited through email invitations from the Center for Medical Simulation’s (CMS) worldwide network of simulation facilitators, former CMS simulation fellows and visiting scholars, and CMS curriculum developers. Sampling was purposeful. All recruited experts gave consent to participate in the study.

Inclusion criteria were:

- Expertise both in debriefing and feedback conversation skills AND teaching them;
- Expertise in both using and teaching advocacy and inquiry as a debriefing and feedback skill;
- 7 years or more of experience as a facilitator in simulation in the healthcare context;
- Can understand and communicate in English;
- That the combination of participants would represent a diversity of professions within healthcare, as well as of the learners with whom they worked.

We assembled a diverse, highly qualified panel to ensure the AIR reflected real-world Advocacy–Inquiry performance across a range of debriefing and feedback contexts. Panel members were experts not only in applying the technique, but also in teaching it and providing feedback on its use—individuals with both tacit, automatic mastery and explicit, deliberative knowledge of the skill. This combination of “doing” and “teaching how to do” aligns with the definition of super-experts in the expertise literature: practitioners who can both perform at a high level and “bystand”—that is, make explicit the reasoning and strategies behind expert performance [38, 39]. Super-experts are uniquely positioned to articulate clear performance criteria. Each panelist had taught at least 20 debriefing or feedback courses, conducted more than 200 debriefing/feedback sessions, participated in at least two such courses as a learner, and brought a minimum of seven years of simulation experience.

The diverse international panel included 39 experts from 12 countries (Supplementary Material 2), capturing cultural and national variance. Professional and teaching role diversity ensured the representation of nurses, midwives, physicians, and social science educators working in settings ranging from medical schools to hospitals, clinics, and rural areas.

This design aimed to provide validity evidence via extrapolation from the test world to real-world applications by linking the AIR assessment to experts' insights across a wide range of contexts. Data were collected anonymously (Supplementary Material 2).

### Delphi method rounds

The four rounds of the Delphi method to elicit and clarify behavioral descriptors related to Advocacy-Inquiry were done anonymously through an online survey system (LimeSurvey V 2.73.1, LimeSurvey GmbH, Germany). All rounds’ questions are gathered in Supplemental Digital Content. Data were stored and managed according to the European General Data Protection Regulation on the University of Caen’s server (France, EU).

#### Round 1

Each expert was prompted to provide unlimited examples of good and poor descriptors for each Advocacy-Inquiry element. Investigators (CB and JWR) qualitatively analyzed responses in two steps: first, we open-coded responses for each element (e.g., 179 responses for “Observation”) to identify a variety of behavioral descriptors [40–42], then we merged descriptors with common themes (e.g., there were 24 occurrences of the theme “orients the listener…” for the Preview, Good, see Table 3) [43]. Author RS reviewed and refined these merged descriptors for clarity. Rankings based on descriptor frequency formed baseline data for Round 2. This round lasted four weeks (two for data collection and two for analysis).

**Table 3:** Qualitatively analyzed responses

	Preview	Occurrence (%)		Rank
Good	Orients the listener to the topic/Describes the topic/Signals a change of topic	24	(86)	1
	Specific: might address who/what/when/where	15	(54)	2
	Is a neutral statement, does not evaluate performance	10	(36)	3
	Is parsimonious, as concise as possible	7	(25)	4
	Seeks permission/Invites to discuss	6	(21)	5
	Facial expression, body language, voice pacing, tone, volume might be reassuring, engaging, calm, or interesting	6	(21)	5
	Provides time estimated for discussion	3	(11)	7
	Describes the speaker's view on cause and effect	2	(7)	8
	Describes the learning objective	2	(7)	8
	Free of improvement suggestion/teaching	1	(4)	10
	Conveys conversation is a joint journey, says "Let's talk about" (instead of "I would like to talk about")	1	(4)	10
	Uses simple, clear terms appropriate to listeners	1	(4)	10
	Invites others to nominate topics	1	(4)	10
Poor	Vague or too broad, non-specific, does not address who/what/when/where	21	(70)	1
	No preview or signal of topic change	14	(47)	2
	Includes a judgment (may be hidden), or an assessment of performance	11	(37)	3
	Is long, is confusing	6	(20)	4
	Facial expression, body language, voice pacing, tone, and volume are threatening, too loud, inappropriate	6	(20)	4
	Misleading preview	5	(17)	6
	Too teacher-centered/objective-based, not matching others' interests	5	(17)	6
	Off-putting words, threatening language	4	(13)	8
	Includes assumptions or inferences	4	(13)	8
	Points out or "calls out" individuals in an unwelcome way	3	(10)	10
	Not distinct from "I saw"	1	(3)	11
	Breaks the fiction contract: e.g. might refer to "the simulation"	1	(3)	11
	I saw/I heard (Observation)	Occurrence (%)		Rank
Good	Describes concrete, visible, audible phenomena or actions, paints a picture	23	(74)	1
	Objective, free of judgment, free of inference	18	(58)	2
	Focused on specific events, might address who/what/when/where	13	(42)	3
	Owns observation as my own, uses "I statements"	8	(26)	4
	Is concise as possible, succinct	7	(23)	5
	Reveals the speaker's areas of uncertainty (e.g. what they didn't hear or see clearly)	6	(19)	6
	Facial expression, body language, voice pacing, tone, volume might be reassuring, engaging, calm, or interesting	6	(19)	6
	Connects to the preview and upcoming "I think"	3	(10)	8
	Not overly rigid in use of "I saw," "I heard", changes phrasing to be authentic	1	(3)	9
	Observations referred to are in the past	1	(3)	9
	Includes the entire team in the description when possible	1	(3)	9
	Uses a short video clip to illustrate	1	(3)	9
Poor	Includes inferences or assumptions about others, ascribes motives, feelings, or thoughts	21	(68)	1
	Vague, too general, too abstract, does not refer to observable phenomena	18	(58)	2
	Includes judgment, critique	12	(39)	3
	Verbal statements are accusatory, and may appear to blame a person or persons	9	(29)	4
	Is long, and covers multiple topics in a way difficult to follow	8	(26)	5
	Not aligned with the facts, incorrect observation	6	(19)	6
	Facial expression, body language, voice pacing, tone, volume could be seen as threatening, too loud, inappropriate	6	(19)	6
	Observation disconnected from the preview, from objectives, from "I think” statement	5	(16)	8
	Does not include an observation, no "I saw/I heard" statement	3	(10)	9
	Presents observations as "the truth", as certain, does not "own" the observation as the speaker's perspective	3	(10)	9
	I think (Point of view)	Occurrence (%)		Rank
Good	Is honest, is "transparent", shares the speaker's judgment, opinion, or assessment	25	(83)	1
	Reveals speakers' reasoning and/or feelings about the link between actions and specific consequences, impacts, implications, effects	23	(77)	2
	Shares perspective as their own, conveys humility	7	(23)	3
	Draws on evidence, guidelines, protocols, best practices	4	(13)	4
	Conveys positive regard, curiosity, respectful interest in others' perspectives	4	(13)	4
	Is parsimonious, is concise as possible, limits the number of topics covered	4	(13)	4
	Facial expression, body language, voice pacing, tone, volume might be reassuring, engaging, calm, or interesting	3	(10)	7
	Connects to the preview, "I saw" in a powerful way	3	(10)	7
	Normalizes the performance (if appropriate)	1	(3)	9
Poor	The speaker's reasoning, judgment, opinion, or take on link between actions and results is missing, implied, cloaked, sugar-coated, or too vague	14	(47)	1
	Unclear, vague, non-specific statements	11	(37)	2
	Verbal statements are accusatory or aggressive, appear to blame or humiliate a person or persons	9	(30)	3
	Includes condemnation of a person or team, mistakes spotlighted as a violation	9	(30)	3
	Speaker omits statements of their point of view completely	7	(23)	5
	Facial expression, body language, voice pacing, tone, volume could be seen as threatening, hostile, too loud, inappropriate	7	(23)	5
	Invokes higher authority, policies, protocols or guidelines without rationale or in a way that seems to force compliance	7	(23)	5
	Not relevant to the subject, lecturing	6	(20)	8
	Includes inferences or assumptions about others, ascribes motives, feelings, or thoughts	6	(20)	8
	Presents own perspective as "the truth", conveys certainty, appears to close off other perspectives	4	(13)	10
	Uses the word "we" when the speaker means "I"	4	(13)	10
	Comments focus on one person when there is shared responsibility	2	(7)	12
	Expressing curiosity, "I am curious" or "I wonder" as the speaker's point of view	1	(3)	13
	Statements not supported by research, protocols, guidelines, incorrect guidance	1	(3)	13
	I wonder (Inquiry)	Occurrence (%)		Rank
Good	Open-ended question that invites a broad range of answers or explanation, is an "essay question"	22	(73)	1
	Conveys genuine curiosity, interest, wonder	15	(50)	2
	Invites listener(s) to share their thinking, reasoning, priorities, frame, values, or perspective, invites them to reflect	13	(43)	3
	Is short, is concise as possible	12	(40)	4
	Facial expression, body language, voice pacing, tone, and volume express interest, are reassuring, engaging	7	(23)	5
	Skillful use of tense, past tense to place the learner back in time, present tense to invite discussion or debate	6	(20)	6
	Free of judgment, inference, teaching, solutions	6	(20)	6
	Only one question	2	(7)	8
	Inquiry links logically to the preview, I saw, I think	2	(7)	8
Poor	Closed-ended, leading, or yes/no question, may start with did/didn't, would/wouldn't, is/isn't, don't you think	19	(63)	1
	Conveys judgment, condemnation, is an inquisition rather than an inquiry	14	(47)	2
	"Guess what I am thinking" question, appears to explore thinking but seeks an answer the speaker has in mind already	9	(30)	3
	Conveys certainty, lacks curiosity	8	(27)	4
	Facial expression, body language, voice pacing, tone, and volume may express exasperation, indignation, disdain, condescension, sarcasm, aggression	8	(27)	4
	Long, confusing, or multi-topic question	8	(27)	4
	Asks for a description of action rather than exploring other person's thinking	5	(17)	7
	"Fix it" or teaching question that invites a change of action, what they would do differently next time	3	(10)	8
	Is a "test" question to assess knowledge (without a preview about the reason for the question)	2	(7)	9
	Includes inferences or assumptions in the question, ascribes motives, feelings, or thoughts	1	(3)	10
	Asking why	1	(3)	10
	Inquiry too narrow	1	(3)	10
	Tense of the question does not elicit needed reflection (i.e. asks a present tense question when past is needed or vice versa)	1	(3)	10
	Listen	Occurrence (%)		Rank
Good	Facial expression, body position, head nods, eye contact, subvocalisations use culturally appropriate cues or mirroring to convey ongoing interest and/or empathy	25	(83)	1
	Paraphrase, reflect, mirror back/repeat or recount what I heard	14	(47)	2
	Internal state: listening intently, is curious, listens to understand	13	(43)	3
	Allows silence	11	(37)	4
	Allow the speaker to finish stating their thoughts, minimize interruptions	11	(37)	4
	Inviting other participants to join the discussion, share their thoughts/Manage the group dynamic	9	(30)	6
	Clarifies or tests own understanding: invites clarification, expansion, deeper explanation	7	(23)	7
	Uses verbal affirmation to encourage others to speak, "Thank you," "I see", " Go on", "Tell me more"	7	(23)	7
	Paraphrases, reflects or mirrors back, repeats back	6	(20)	9
	The ability to read their response, not only the tone of voice by but body posture as well	3	(10)	10
	Synthesizes, elevates, links disparate concepts together for the group's benefit	3	(10)	10
	Verbally conveys empathy and normalizes challenges	2	(7)	12
Poor	Interrupts, talks over, cuts people off too often	22	(73)	1
	Facial expression, body position, head nods or position, direction of gaze, subvocalisations convey distraction, disinterest, impatience, inappropriate focus	17	(57)	2
	Facial expression, body position, head shaking, hand waving, eye-rolling convey contempt, anger, distaste, disdain, skepticism, suspicion	12	(40)	3
	Changing or pivoting to a new subject without acknowledging what was said, not acknowledging what was said	12	(40)	3
	Voice tone, words, or paravocal sounds (sighing, sniffing, grunting, harsh laughter, tongue clicking, muttering under one's breath) convey disdain, condemnation, anger, suspicion	8	(27)	5
	Dismissing other person's worries, concerns, focus	4	(13)	6
	Arguing in a way that suppresses other person's sharing their point of view	4	(13)	6
	Correcting or interpreting other people's thoughts in a way that suppresses their talking	3	(10)	8
	Fills the silence, does not leave silence, does not respect the silence	3	(10)	8
	Lecturing or talking ad nauseam	3	(10)	8
	No paraphrasing or checking back with the other person to verify what was heard	2	(7)	11
	Internal state: listening to respond, listening for the "right" answer, planning the next statement	2	(7)	11
	No verbal encouragement for others to speak	1	(3)	13

#### Round 2

Experts rated descriptors from Round 1 on a 6-point Likert importance scale. We calculated means and standard deviations, ranking descriptors by importance, using Round 1 rankings to break ties. The top three descriptors were classified as “high-rated,” the next four as “average-rated,” and the remainder as “lowest-rated.” This round lasted three weeks (two for data collection and one for analysis).

#### Round 3

Experts reviewed descriptors from Round 2 to confirm whether to keep the “high-rated” ones and discard the “low-rated” ones. They were also asked to rank the “average-rated” ones according to their importance. The aim was to narrow the list of descriptors to six per element for adaptive learning and assessment. “Highly-rated” descriptors with ≥ 85% agreement were confirmed. The top three “average-rated” descriptors, weighted by ranking, were retained. “Lowest-rated” descriptors were discarded if ≥ 55% of experts agreed to remove them. Near-final descriptor lists were created. This round lasted 10 days (eight for data collection and two for analysis).

#### Round 4

Experts evaluated near-final descriptor lists with two questions per descriptor: Should it be included? If yes, at what practitioner level (beginner, intermediate, advanced)? Weighted scoring determined the classification: beginner (lowest scores), advanced (highest scores), and intermediate (middle scores). Final lists of descriptors aimed to support adaptive learning and assessment, with two descriptors per skill level, allowing users to choose 2 to 6 descriptors to adapt to the cognitive load. This round lasted two weeks (one for data collection and one for analysis).

### Developing the scales and usability testing

**Fig. 2:**
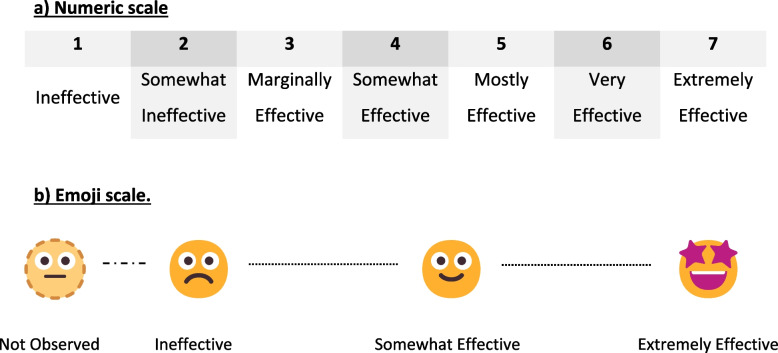
Description of the anchored rating scales for the Advocacy-Inquiry Rubric (AIR)

To develop the **numeric scale**, we drew on the author team’s research on and experience with the Debriefing Assessment for Simulation in Healthcare (DASH). The numeric AIR combines behavioral descriptors with numeric ratings to enhance usability and clarity [2, 44]. To give facilitators a clear developmental path toward excellent performance, the seven-point scale includes two unacceptable ratings (1 and 2) and five acceptable levels, allowing finer distinctions in positive performance [45] (see Fig. 2a).

Usability testing with two international simulation facilitator cohorts informed graphic display revisions that linked good and poor behavioral anchors to clarify whether behaviors exist on a continuum or independently (see Supplementary Material 3). We conducted a second series of tests and group interviews with simulation facilitator cohorts and asked them to imagine using the scale at their home programs to introduce the AIR or to provide peer feedback.

During usability testing with three separate facilitator cohorts over three months, participants applied the draft versions of the numeric AIR to debriefing video excerpts and subsequently participated in focus groups with the author (JWR). The focus groups explored the clarity of descriptors, ease of use, and perceived applicability to formative and summative contexts. Feedback was analyzed qualitatively by the study team to identify recurrent suggestions. The usability testing inspired the development of the **emoji scale** (Fig. 2b). When asked to imagine using the numeric scale at their home programs, facilitators-in-training expressed discomfort with giving numeric ratings to peers. We also identified the value of broad distinctions in performance for peer feedback: problematic, okay, good. From these insights, we developed the three-point emoji scale. Therefore, we wanted to test the emoji scale first because such a scale currently does not exist in the feedback or debriefing literature. We report the validity evidence for this test.

For reliability testing, three independent raters used the finalized numeric AIR to score recorded debriefing segments, each corresponding to a distinct facilitator. Inter-rater reliability was calculated using a two-way random-effects model, absolute agreement, Intraclass Correlation Coefficient (ICC), with 95% confidence intervals. Sample size and number of ratings were selected to meet recommended thresholds for stable ICC estimates.

## Results

Demographic data for the expert panel is summarized in Supplementary Material 3.

### Delphi study

**Fig. 3:**
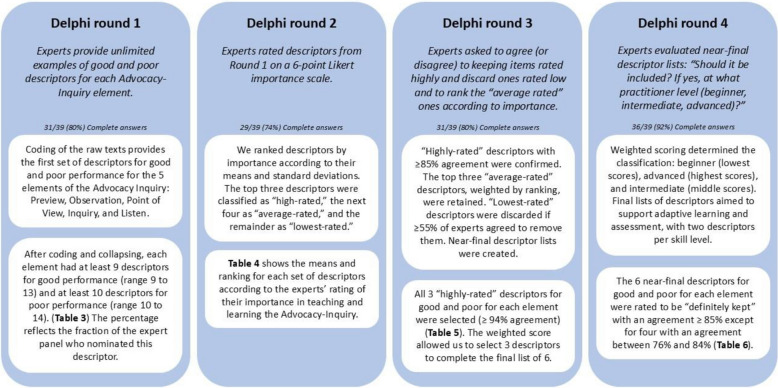
Main steps and results of the four Delphi rounds

The study, conducted over 10 weeks between April and June 2019, included 39 experts of the 41 solicited in 4 rounds of the Delphi method. We requested answers from every expert for every round. Of the 39 experts who answered, 3 answered one round, 4 answered two rounds, 12 answered three rounds, and 20 answered all the rounds. Figure 3 summarizes the main steps and results of the four Delphi rounds.

#### Round 1

The coding of the raw texts of the experts’ answers provided the first set of descriptors for good and poor performance for the 5 elements of the Advocacy Inquiry: Preview, Observation, Point of View, Inquiry, and Listen (Table 3). A total of 871 items were collected and then merged into 119 descriptors for the five elements (good and poor). After coding and collapsing, each element had at least 9 descriptors for good performance (range 9 to 13) and at least 10 descriptors for poor performance (range 10 to 14). The percentage reflects the fraction of the expert panel who nominated this descriptor.

#### Round 2

Table 4 shows the means and ranking for each set of descriptors according to the experts’ rating of their importance in teaching and learning Advocacy-Inquiry. The comparison of rounds 1 and 2 rankings shows that the most frequently cited in round 1 were ranked among the 4 most important for every good and poor descriptor set in round 2, except for the *poor preview*. This comparison also shows that some descriptors rarely nominated in round 1 were rated highly important in round 2. An example is “Uses simple, clear terms appropriate to listeners” (Preview, Good), nominated only one time in Round 1 and rated 4.9, and ranked second in Round 2.

**Table 4:** The means and ranking for each set of descriptors

	Preview	Mean	SD	Rank	Rank (R1)
Good	Orients the listener to topic/Describes the topic/Signals a change of topic	5,7	0,5	1	1
	Is a neutral statement, does not evaluate performance	4,9	1,1	2	3
	Uses simple, clear terms appropriate to listeners	4,9	0,8	2	10
	Facial expression, body language, voice pacing, tone, volume might be reassuring, engaging, calm, or interesting	4,8	1,4	4	5
	Seeks permission/Invites to discuss	4,3	1,1	5	5
	Free of improvement suggestion/teaching	4,3	1,6	5	10
	Specific: might address who/what/when/where	4,2	1,4	7	2
	Is parsimonious, as concise as possible	4,1	1,2	8	4
	Conveys conversation is a joint journey, says "Let's talk about" (instead of "I would like to talk about")	3,9	1,5	9	10
	Provides time estimated for discussion	3,5	1,6	10	7
	Invites others to nominate topics	3,4	1,5	11	10
	Describes the learning objective	3,3	1,7	12	8
	Describes the speaker's view on cause and effect	2,4	1,7	13	8
Poor	No preview or signal of topic change	5,7	1,2	1	2
	Off-putting words, threatening language	5,6	1,1	2	8
	Misleading preview	5,5	1,2	3	6
	Points out or "calls out" individuals in an unwelcome way	5,5	1,1	3	10
	Includes a judgment (may be hidden), or an assessment of performance	5,4	1,2	5	3
	Facial expression, body language, voice pacing, tone, and volume are threatening, too loud, inappropriate	5,3	1,2	6	4
	Includes assumptions or inferences	5,2	1,2	7	8
	Vague or too broad, non-specific, does not address who/what/when/where	5,1	1,4	8	1
	Is long, is confusing	4,9	1,2	9	4
	Not distinct from "I saw"	4,6	1,7	10	11
	Too teacher-centered/objective-based, not matching others' interests	4,3	1,7	11	6
	Breaks the fiction contract: e.g. might refer to "the simulation"	3,7	1,9	12	11
	I saw / I heard (Observation)	Mean	SD	Rank	Rank (R1)
Good	Describes concrete, visible, audible phenomena or actions, paints a picture	5,8	1,7	1	1
	Objective, free of judgment, free of inference	5,6	1,7	2	2
	Owns observation as my own, uses "I statements"	5,5	1,7	3	4
	Focused on specific events, might address who/what/when/where	5,3	1,7	4	3
	Connects to the preview, and upcoming "I think"	5,2	1,6	5	8
	Facial expression, body language, voice pacing, tone, volume might be reassuring, engaging, calm, or interesting	4,9	1,7	6	6
	Reveals the speaker's areas of uncertainty (e.g. what they didn't hear or see clearly)	4,7	1,8	7	6
	Is concise as possible, succinct	4,4	1,6	8	5
	Not overly rigid in use of "I saw," "I heard", changes phrasing to be authentic	4,3	1,5	9	9
	Observations referred to are in the past	3,9	1,7	10	9
	Includes the entire team in the description when possible	3,5	1,5	11	9
	Uses a short video clip to illustrate	2,6	1,4	12	9
Poor	Verbal statements are accusatory, may appear to blame a person or persons	5,8	1,7	1	4
	Vague, too general, too abstract, does not refer to observable phenomena	5,7	1,7	2	2
	Includes judgment, critique	5,6	1,9	3	3
	Includes inferences or assumptions about others, ascribes motives, feelings, or thoughts	5,5	1,7	4	1
	Not aligned with the facts, incorrect observation	5,5	1,9	4	6
	Facial expression, body language, voice pacing, tone, volume could be seen as threatening, too loud, inappropriate	5,5	1,7	4	6
	Does not include an observation, no "I saw/I heard" statement	5,4	1,7	7	9
	Presents observations as "the truth", as certain, does not "own" the observation as the speaker's perspective	5,4	1,7	7	9
	Is long, covers multiple topics in a way difficult to follow	5,3	1,7	9	5
	Observation disconnected from the preview, from objectives, from "I think” statement	5,1	1,6	10	8
	I think (Point of view)	Mean	SD	Rank	Rank (R1)
Good	Reveals speakers' reasoning and/or feelings about the link between actions and specific consequences, impacts, implications, effects	5,8	1,9	1	2
	Is honest, is "transparent", shares the speaker's judgment, opinion, or assessment	5,7	1,9	2	1
	Shares perspective as their own, conveys humility	5,6	1,9	3	3
	Conveys positive regard, curiosity, respectful interest in others' perspectives	5,4	1,9	4	4
	Connects to the preview, "I saw" in a powerful way	5,1	1,9	5	7
	Facial expression, body language, voice pacing, tone, volume might be reassuring, engaging, calm, or interesting	5,0	2,0	6	7
	Is parsimonious, is concise as possible, limits the number of topics covered	4,3	1,8	7	4
	Normalizes the performance (if appropriate)	4,3	1,7	7	9
	Draws on evidence, guidelines, protocols, best practices	4,2	1,9	9	4
Poor	Verbal statements are accusatory or aggressive, appear to blame or humiliate a person or persons	5,8	1,9	1	3
	Includes condemnation of a person or team, mistakes spotlighted as a violation	5,8	1,9	2	3
	The speaker's reasoning, judgment, opinion, or take on link between actions and results is missing, implied, cloaked, sugar-coated, or too vague	5,7	1,9	3	1
	Presents own perspective as "the truth", conveys certainty, appears to close off other perspectives	5,6	5,4	4	10
	Unclear, vague, non-specific statements	5,4	1,9	5	2
	Speaker omits statements of their point of view completely	5,4	1,9	5	5
	Facial expression, body language, voice pacing, tone, volume could be seen as threatening, hostile, too loud, inappropriate	5,4	1,9	5	5
	Not relevant with the subject, lecturing	5,1	2	8	8
	Includes inferences or assumptions about others, ascribes motives, feelings, or thoughts	5,1	2	8	8
	Invokes higher authority, policies, protocols or guidelines without rationale or in a way that seems to force compliance	5	2	10	5
	Comments focus on one person when there is shared responsibility	4,9	1,8	11	12
	Uses the word "we" when the speaker means "I"	4,8	2	12	10
	Statements not supported by research, protocols, guidelines, incorrect guidance	4,6	2,1	13	13
	Expressing curiosity, "I am curious" or "I wonder" as the speaker's point of view	3,8	2,3	14	13
	I wonder (Inquiry)	Mean	SD	Rank	Rank (R1)
Good	Invites listener(s) to share their thinking, reasoning, priorities, frame, values, or perspective, invites them to reflect	5,8	1,9	1	3
	Conveys genuine curiosity, interest, wonder	5,7	1,9	2	2
	Open-ended question that invites a broad range of answers or explanation, is an "essay question"	5,4	1,9	3	1
	Inquiry links logically to the preview, I saw, I think	5,3	1,9	4	8
	Facial expression, body language, voice pacing, tone, and volume express interest, are reassuring, engaging	5,2	1,9	5	5
	Free of judgment, inference, teaching, solutions	5,2	1,8	5	6
	Is short, is concise as possible	5,1	1,8	7	4
	Only one question	4,8	1,8	8	8
	Skillful use of tense, past tense to place the learner back in time, present tense to invite discussion or debate	4,4	1,8	9	6
Poor	Closed-ended, leading, or yes/no question, may start with did/didn't, would/wouldn't, is/isn't, don't you think	5,7	1,9	1	1
	Conveys judgment, condemnation, is an inquisition rather than an inquiry	5,7	1,9	1	2
	"Guess what I am thinking" question, appears to explore thinking but seeks an answer the speaker has in mind already	5,5	1,9	3	3
	Facial expression, body language, voice pacing, tone, and volume may express exasperation, indignation, disdain, condescension, sarcasm, aggression	5,5	2,1	3	4
	Conveys certainty, lacks curiosity	5,4	1,9	5	4
	Includes inferences or assumptions in the question, ascribes motives, feelings, or thoughts	5,3	1,8	6	10
	Is a "test" question to assess knowledge (without a preview about the reason for the question)	5,2	1,8	7	9
	Long, confusing, or multi-topic question	5,1	1,8	8	4
	Inquiry not preceded by preview or advocacy, "naked inquiry"	5,1	1,9	8	14
	Asks for a description of action rather than exploring other person's thinking	5,1	2	8	7
	"Fix it" or teaching question that invites a change of action, what they would do differently next time	4,9	1,8	11	8
	Tense of the question does not elicit needed reflection (i.e. asks a present tense question when past is needed or vice versa)	4,3	1,9	12	10
	Inquiry too narrow	4,1	2	13	10
	Asking why	3,8	2,1	14	10
	Listen	Mean	SD	Rank	Rank (R1)
Good	Facial expression, body position, head nods, eye contact, subvocalisations use culturally appropriate cues or mirroring to convey on-going interest and/or empathy	5,6	1,9	1	1
	Allows silence	5,5	1,9	2	4
	Internal state: listening intently, is curious, listens to understand	5,4	2	3	3
	Allow the speaker to finish stating their thoughts, minimize interruptions	5,3	1,9	4	4
	Uses verbal affirmation to encourage others to speak, "Thank you," "I see", " Go on", "Tell me more"	5,2	1,8	5	7
	Clarifies or tests own understanding: invites clarification, expansion, deeper explanation	5,1	1,8	6	7
	Verbally conveys empathy and normalize challenges	5,1	1,9	6	12
	Paraphrase, reflect, mirror back/repeat or recount what I heard	5	1,9	8	2
	Inviting other participants to join the discussion, share their thoughts / Manage the group dynamic	5	1,9	8	6
	The ability to read their response, not only the tone of voice by but body posture as well.	5	1,9	8	10
	Synthesizes, elevates, links disparate concepts together for the group's benefit	5	1,9	8	10
Poor	Interrupts, talks over, cuts people off too often	5,8	1,9	1	1
	Facial expression, body position, head nods or position, direction of gaze, subvocalisations convey distraction, disinterest, impatience, inappropriate focus	5,7	1,9	2	2
	Facial expression, body position, head shaking, hand waving, eye-rolling convey contempt, anger, distaste, disdain, skepticism, suspicion	5,7	1,9	2	3
	Voice tone, words, or paravocal sounds (sighing, sniffing, grunting, harsh laughter, tongue clicking, muttering under one's breath) convey disdain, condemnation, anger, suspicion	5,7	1,9	2	5
	Arguing in a way that suppresses other person's sharing their point of view	5,7	1,9	2	6
	Dismissing other person's worries, concerns, focus	5,6	1,9	6	6
	Correcting or interpreting other people's thoughts in a way that suppresses their talking	5,6	2,1	6	8
	Lecturing or talking ad nausea	5,5	1,9	8	8
	Fills the silence, does not leave silence, does not respect the silence	5,4	2	9	8
	Changing or pivoting to a new subject without acknowledging what was said, not acknowledging what was said	5,3	1,9	10	3
	Internal state: listening to respond, listening for the "right" answer, planning the next statement	5,2	2	11	11
	No paraphrasing or checking back with the other person to verify what was heard	5,1	2	12	11
	Jumping to share a "solution" or fix	4,9	1,8	13	13
	No verbal encouragement for others to speak	4,8	1,9	14	13

#### Round 3

All 3 “high-rated descriptors” at round 2 for good and poor for each element were selected if they had at least 94% agreement (except one which was kept with 87% agreement: Listen, Poor “*Voice tone, words, or paravocal sounds (sighing, sniffing, grunting, harsh laughter, tongue clicking, muttering under one's breath) convey disdain, condemnation, anger, suspicion*” (Table 5). The weighted score built on the second ranking of importance (round 3) among the 4 “average-rated descriptors” allowed us to identify and select 3 descriptors to complete the final list of 6 descriptors for each good and poor set for the 5 elements. The percentages of agreement to discard the lowest descriptors were all ≥ 55% (from 56 to 91%). There were more agreements on the descriptors to keep than on the ones to discard.

**Table 5:** Near-final list of 6 descriptors for each good and poor set for the 5 elements

	Preview	Keep/Discard (%)		Rank 1	Rank 2		Rank 3	Rank 4	Score	
Good	Orients the listener to topic/Describes the topic/Signals a change of topic	32	(100)							
	Is a neutral statement, does not evaluate performance	31	(97)							
	Uses simple, clear terms appropriate to listeners	31	(97)							
	Specific: Might address who/what/when/where			11	7	8		6	87	
	Is parsimonious, as concise as possible			9	6	9		8	80	
	Seeks permission/Invites to discuss			7	10	5		10	78	
	Free of improvement suggestion/teaching			5	9	10		8	75	
	Conveys conversation is a joint journey, says "Let's talk about" (instead of "I would like to talk about")	24	(75)							
	Provides time estimated for discussion	27	(84)							
	Invites others to nominate topics	28	(88)							
	Describes the learning objective	25	(76)							
	Describes the speaker's view on cause and effect	29	(91)							
Poor	No preview or signal of topic change	31	(97)							
	Off-putting words, threatening language	31	(97)							
	Misleading preview	31	(97)							
	Points out or "calls out" individuals in an unwelcome way			18	5	4		5	100	
	Includes assumptions or inferences			5	9	14		4	79	
	Includes a judgment (may be hidden), or an assessment of performance			3	12	9		8	74	
	Vague or too broad, non-specific, does not address who/what/when/where			6	6	5		15	67	
	Is long, is confusing	18	(56)							
	Not distinct from "I saw"	27	(84)							
	Too teacher-centered/objective-based, no matching others' interests	25	(78)							
	Breaks the fiction contract: e.g. might refer to "the simulation"	29	(91)							
	I saw / I heard (Observation)	Keep/Discard (%)		Rank 1	Rank 2		Rank 3	Rank 4	Score	
Good	Describes concrete, visible, audible phenomena or actions, paints a picture	32	(100)							
	Objective, free of judgment, free of inference	32	(100)							
	Owns observation as my own, uses "I statements"	32	(100)							
	Focused on specific events, might address who/what/when/where			11	10	8		3	93	
	Connects to the preview and upcoming "I think"			10	11	7		4	91	
	Reveals the speaker's areas of uncertainty (e.g. what they didn't hear or see clearly)			8	7	9		8	79	
	Is concise as possible, succinct			3	4	8		17	57	
	Not overly rigid in use of "I saw," "I heard", changes phrasing to be authentic	24	(75)							
	Observations referred to are in the past	26	(81)							
	Includes entire team in the description when possible	28	(88)							
	Uses a short video clip to illustrate	23	(72)							
Poor	Verbal statements are accusatory, may appear to blame a person or persons	31	(97)							
	Vague, too general, too abstract, does not refer to observable phenomena	31	(97)							
	Includes judgment, critique	31	(97)							
	Presents observations as "the truth", as certain, does not "own" the observation as the speaker's perspective			8	13	6		5	88	
	Does not include an observation, no "I saw/I heard" statement			12	6	6		8	86	
	Includes inferences or assumptions about others, ascribes motives, feelings, or thoughts			8	6	10		8	78	
	Not aligned with the facts, incorrect observation			4	7	10		11	68	
	Is long, covers multiple topics in a way difficult to follow	19	(59)							
	Observation disconnected from the preview, from objectives, from "I think” statement	21	(66)							
	I think (Point of view)	Keep/Discard (%)		Rank 1	Rank 2		Rank 3	Rank 4	Rank 5	Score
Good	Reveals speakers' reasoning and/or feelings about the link between actions and specific consequences, impacts, implications, effects	31	(97)							
	Is honest, is "transparent", shares the speaker's judgment, opinion, or assessment	32	(100)							
	Shares perspective as their own, conveys humility	31	(97)							
	Conveys positive regard, curiosity, respectful interest in others' perspectives			18	10	3		1	-	109
	Connects to the preview, "I saw" in a powerful way			7	11	10		4	-	85
	Normalizes the performance (if appropriate)			4	8	10		10	-	70
	Is parsimonious, is concise as possible, limits the number of topics covered			3	3	9		17	-	56
	Draws on evidence, guidelines, protocols, best practices	20	(63)							
Poor	Verbal statements are accusatory or aggressive, appear to blame or humiliate a person or persons	31	(97)							
	Includes condemnation of a person or team, mistakes spotlighted as a violation	31	(97)							
	The speaker's reasoning, judgment, opinion, or take on link between actions and results is missing, implied, cloaked, sugar-coated, or too vague	32	(100)							
	Includes inferences or assumptions about others, ascribes motives, feelings, or thoughts			11	10	6		4	1	122
	Presents own perspective as "the truth", conveys certainty, appears to close off other perspectives			9	10	4		4	5	110
	Speaker omits statements of their point of view completely			10	5	7		6	4	107
	Unclear, vague, non-specific statements			0	4	9		11	8	73
	Not relevant with the subject, lecturing			2	3	6		7	14	68
	Invokes higher authority, policies, protocols or guidelines without rationale or in a way that seems to force compliance	20	(63)							
	Comments focus on one person when there is shared responsibility	20	(63)							
	Uses the word "we" when the speaker means "I"	21	(64)							
	Statements not supported by research, protocols, guidelines, incorrect guidance	20	(63)							
	Expressing curiosity, "I am curious" or "I wonder" as the speaker's point of view	25	(78)							
	I wonder (Inquiry)	Keep/Discard (%)		Rank 1	Rank 2		Rank 3	Rank 4	Score	
Good	Invites listener(s) to share their thinking, reasoning, priorities, frame, values, or perspective, invites them to reflect	31	(100)							
	Conveys genuine curiosity, interest, wonder	29	(94)							
	Open-ended question that invites a broad range of answers or explanation, is an "essay question"	30	(97)							
	Free of judgment, inference, teaching, solutions			12	10	3		6	90	
	Is short, is concise as possible			10	4	12		5	81	
	Inquiry links logically to the preview, I saw, I think			4	13	5		9	74	
	Only one question			5	4	11		11	65	
	Skillful use of tense, past tense to place the learner back in time, present tense to invite discussion or debate	25	(81)							
Poor	Closed-ended, leading, or yes/no question, may start with did/didn't, would/wouldn't, is/isn't, don't you think	31	(100)							
	Conveys judgment, condemnation, is an inquisition rather than inquiry	30	(97)							
	"Guess what I am thinking" question, appears to explore thinking but seeks an answer the speaker has in mind already	31	(100)							
	Includes inferences or assumptions in the question, ascribes motives, feelings, or thoughts			10	11	5		5	88	
	Is a "test" question to assess knowledge (without a preview about the reason for the question)			9	9	8		5	84	
	Conveys certainty, lacks curiosity			7	6	9		9	73	
	Long, confusing, or multi-topic question			5	5	9		12	65	
	Inquiry not preceded by preview or advocacy, "naked inquiry"	20	(65)							
	Asks for a description of action rather than exploring other person's thinking	21	(68)							
	"Fix it" or teaching question that invites a change of action, what they would do differently next time	18	(58)							
	Tense of question does not elicit needed reflection (i.e. asks a present tense question when past is needed or vice versa)	25	(81)							
	Inquiry too narrow	28	(90)							
	Asking why	25	(81)							
	Listen	Keep/Discard (%)		Rank 1	Rank 2		Rank 3	Rank 4	Score	
Good	Allows silence	31	(100)							
	Internal state: listening intently, is curious, listens to understand	29	(94)							
	Allow the speaker to finish stating their thoughts, minimizes interruptions	31	(100)							
	Clarifies or tests own understanding: invites clarification, expansion, deeper explanation			11	10	7		3	91	
	Uses verbal affirmation to encourage others to speak, "Thank you," "I see", " Go on", "Tell me more"			7	10	9		5	81	
	Paraphrase, reflect, mirror back/repeat or recount what I heard			6	8	11		6	76	
	Verbally conveys empathy and normalizes challenges			7	3	4		17	62	
	Inviting other participants to join the discussion, share their thoughts / Manage the group dynamic	18	(58)							
	The ability to read their response, not only the tone of voice by but body posture as well.	22	(71)							
	Synthesizes, elevates, links disparate concepts together for the group's benefit	21	(68)							
Poor	Interrupts, talks over, cuts people off too often	31	(100)							
	Voice tone, words, or paravocal sounds (sighing, sniffing, grunting, harsh laughter, tongue clicking, muttering under one's breath) convey disdain, condemnation, anger, suspicion	27	(87)							
	Arguing in a way that suppresses other person's sharing their point of view	31	(100)							
	Correcting or interpreting other people's thoughts in a way that suppresses their talking			12	9	6		4	91	
	Dismissing other person's worries, concerns, focus			10	12	4		5	89	
	Lecturing or talking ad nauseam			3	8	13		7	69	
	Fills the silence, does not leave silence, does not respect the silence			6	2	8		15	61	
	Changing or pivoting to a new subject without acknowledging what was said, not acknowledging what was said	18	(58)							
	Internal state: listening to respond, listening for the "right" answer, planning next statement	19	(61)							
	No paraphrasing or checking back with the other person to verify what was heard	20	(65)							
	Jumping to share a "solution" or fix	19	(61)							
	No verbal encouragement for others to speak	21	(68)							

#### Round 4

The 6 near-final descriptors for good and poor for each element, selected from round 3, were rated to be “definitely kept” with an agreement ≥ 85% except for four with an agreement between 76 and 84%: Preview, good “*Is parsimonious, as concise as possible*.” (76%), Preview, good “*Specific: Might address who/what/when/where*.” (81%), Preview, good “*Seeks permission/Invites to discuss*.” (84%), and Observation, good “*Reveals speaker's areas of uncertainty (e.g. what they didn't hear or see clearly)*.” (84%) (Table 6). Given the proximity of the scores obtained to the 85% limit and the limited number of descriptors concerned (4 out of 60), the investigators (CB, JWR, RS) decided not to carry out a fifth round of Delphi and to select the final list of descriptors as it stood. The lower agreements to keep the descriptors were more frequent in the descriptors considered as advanced.

**Table 6:** Final list of 6 descriptors for each good and poor set for the 5 elements, with beginner, intermediate and advanced allocation

	Preview	Keep (%)			Beginner	Intermediate	Advanced	Score
Good	Orients the listener to the topic/Describes the topic/Signals a change of topic	37		(100)	29	5	3	1.30
	Uses simple, clear terms appropriate to listeners	37		(100)	19	15	3	1.57
	Is a neutral statement, does not evaluate performance	36		(97)	17	17	3	1.62
	Specific: Might address who/what/when/where	30		(81)	14	15	8	1.84
	Seeks permission/Invites to discuss	31		(84)	16	10	11	1.86
	Is succinct, as concise as possible	28		(76)	7	14	16	2.24
Poor	Off-putting words, threatening language	37		(100)	29	5	3	1.16
	No preview or signal of topic change	37		(100)	19	15	3	1.24
	Points out or "calls out" individuals in an unwelcome way	36		(97)	17	17	3	1.30
	Misleading preview	30		(81)	14	15	8	1.59
	Includes assumptions or inferences	31		(84)	16	10	11	1.84
	Includes a judgment (may be hidden), or an assessment of performance	28		(76)	7	14	16	1.97
	I saw / I heard (Observation)	Keep (%)			Beginner	Intermediate	Advanced	Score
Good	Describes concrete, visible, audible phenomena or actions, paints a picture	36	(97)		24	12	1	1.38
	Owns observation as my own, uses "I statements"	37	(100)		24	11	2	1.41
	Focused on specific events, might address who/what/when/where	36	(97)		19	15	3	1.57
	Objective, free of judgment, free of inference	37	(100)		14	20	3	1.70
	Connects to the preview and upcoming "I think"	37	(100)		9	22	6	1.92
	Reveals the speaker's areas of uncertainty (e.g. what they didn't hear or see clearly)	31	(84)		6	16	15	2.24
Poor	Does not include an observation, no "I saw/I heard" statement	37	100)		32	4	1	1.16
	Verbal statements are accusatory; may appear to blame a person or persons	36	(97)		30	7	0	1.19
	Vague, too general, too abstract, does not refer to observable phenomena	37	(100)		20	17	0	1.46
	Includes judgment, critique	36	(97)		17	17	3	1.62
	Presents observations as "the truth", as certain, does not "own" the observation as the speaker's perspective	36	(97)		15	15	7	1.78
	Includes inferences or assumptions about others, ascribes motives, feelings, or thoughts	37	(100)		10	22	5	1.86
	I think (Point of view)	Keep (%)			Beginner	Intermediate	Advanced	Score
Good	Is honest, is "transparent", shares the speaker's judgment, opinion, or assessment	37	(100)		19	17	1	1.51
	Shares perspective as their own; conveys humility	36	(97)		18	16	3	1.59
	Conveys positive regard, curiosity, respectful interest in others' perspectives	33	(89)		17	12	8	1.76
	Reveals speakers' reasoning and/or feelings about the link between actions and specific consequences, impacts, implications, effects	36	(97)		14	14	9	1.86
	Connects to the preview, "I saw" in a powerful way	35	(95)		6	20	11	2.14
	Normalizes the performance (if appropriate)	32	(86)		5	21	11	2.16
Poor	Verbal statements are accusatory or aggressive; appear to blame or humiliate a person or persons	37	(100)		32	5	0	1.14
	Includes condemnation of a person or team, mistakes spotlighted as a violation	36	(97)		31	5	1	1.19
	Speaker omits statements of their point of view completely	36	(97)		28	8	1	1.27
	The speaker's reasoning, judgment, opinion, or take on the link between actions and results is missing, implied, cloaked, sugar-coated, or too vague	36	(97)		10	24	3	1.81
	Presents own perspective as "the truth", conveys certainty, appears to close off other perspectives	36	(97)		15	14	8	1.81
	Includes inferences or assumptions about others, ascribes motives, feelings, or thoughts	36	(97)		12	17	8	1.89
	I wonder (Inquiry)	Keep (%)			Beginner	Intermediate	Advanced	Score
Good	An open-ended question that invites a broad range of answers or explanations, is an "essay question"	37	(100)		23	12	2	1.43
	Free of judgment, inference, teaching, solutions	37	(100)		19	16	2	1.54
	Invites listener(s) to share their thinking, reasoning, priorities, frame, values, or perspective, invites them to reflect	37	(100)		19	14	4	1.59
	Inquiry links logically to the preview, I saw, I think	33	(89)		14	17	6	1.78
	Is short, is concise as possible	34	(92)		16	13	8	1.78
	Conveys genuine curiosity, interest, wonder	34	(92)		16	11	10	1.84
Poor	Closed-ended, leading, or yes/no question, may start with did/didn't, would/wouldn't, is/isn't, don't you think	37	(100)		27	9	1	1.30
	Conveys judgment, condemnation, is an inquisition rather than an inquiry	37	(100)		23	14	0	1.38
	Is a "test" question to assess knowledge (without a preview about the reason for the question)	37	(100)		23	12	2	1.43
	Conveys certainty, lacks curiosity	36	(97)		21	15	1	1.46
	"Guess what I am thinking" question, appears to explore thinking but seeks an answer the speaker has in mind already	37	(100)		13	22	2	1.70
	Includes inferences or assumptions in the question, ascribes motives, feelings, or thoughts	37	(100)		16	16	5	1.70
	Listen	Keep (%)			Beginner	Intermediate	Advanced	Score
Good	Allow the speaker to finish stating their thoughts, minimize interruptions	37	(100)		25	11	1	1.35
	Uses verbal affirmation to encourage others to speak, "Thank you," "I see", " Go on", "Tell me more"	37	(100)		23	14	0	1.38
	Paraphrase, reflect, mirror back/repeat or recount what I heard	33	(89)		15	14	8	1.81
	Internal state: listening intently, is curious, listens to understand	33	(89)		12	15	10	1.95
	Allows silence	37	(100)		9	19	9	2.00
	Clarifies or tests own understanding: invites clarification, expansion, deeper explanation	37	(100)		7	23	7	2.00
Poor	Interrupts, talks over, cuts people off too often	35	(97)		26	10	0	1.28
	Voice tone, words, or paravocal sounds (sighing, sniffing, grunting, harsh laughter, tongue clicking, muttering under one's breath) convey disdain, condemnation, anger, suspicion	35	(97)		26	7	3	1.36
	Arguing in a way that suppresses other person's sharing their point of view	36	(100)		19	15	2	1.53
	Lecturing or talking ad nauseam	36	(100)		19	14	3	1.56
	Dismissing other person's worries, concerns, focus	36	(100)		17	14	5	1.67
	Correcting or interpreting other people's thoughts in a way that suppresses their talking	36	(100)		13	19	4	1.75

The weighted average score built on the level of expertise expected from a practitioner (beginner, intermediate, or advanced) allowed us to rank the descriptors in each set from the “easiest” ones (lower score) to the more advanced (higher score) with 2 descriptors for each beginner, intermediate, and advanced (Table 6). Two similar scoring situations between intermediate and advanced occurred for Point of View, poor, between “*Presents own perspective as "the truth", conveys certainty, appears to close off other perspectives*” and “*The speaker's reasoning, judgment, opinion, or take on the link between actions and results is missing, implied, cloaked, sugar-coated, or too vague*”; and for Inquiry, good, between “*Inquiry links logically to the preview, I saw, I think*” and “*Is short, is concise as possible*”. The investigators (CB, JWR, RS) separated descriptors with similar scores based on the number of “intermediate” and “advanced” occurrences, with the aim of maintaining an adaptive learning and assessment approach with two descriptors per skill level. This was deemed acceptable, as it did not modify the selection of descriptors, but only the order of appearance of 4 descriptors out of 60 in the progressive gradation of the difficulty level.

#### Rubric

**Fig. 4:**
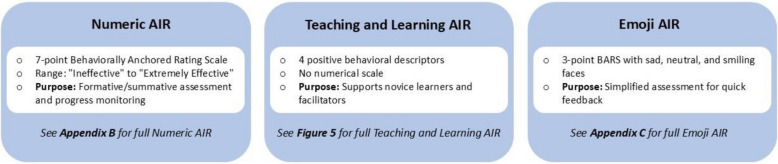
The three Advocacy-Inquiry Rubrics’ (AIR) main features

Our Delphi research identified six positive and six negative performance descriptors for each of the five Advocacy–Inquiry elements: Preview, Observation, Point of View, Inquiry, and Listen. Two beginner descriptors were deemed foundational skills, two intermediate, and two advanced descriptors built on that. We embedded these behavioral descriptors in three versions of the AIR. The features that differentiate the three versions of AIR are presented in Fig. 4.

**Fig. 5:**
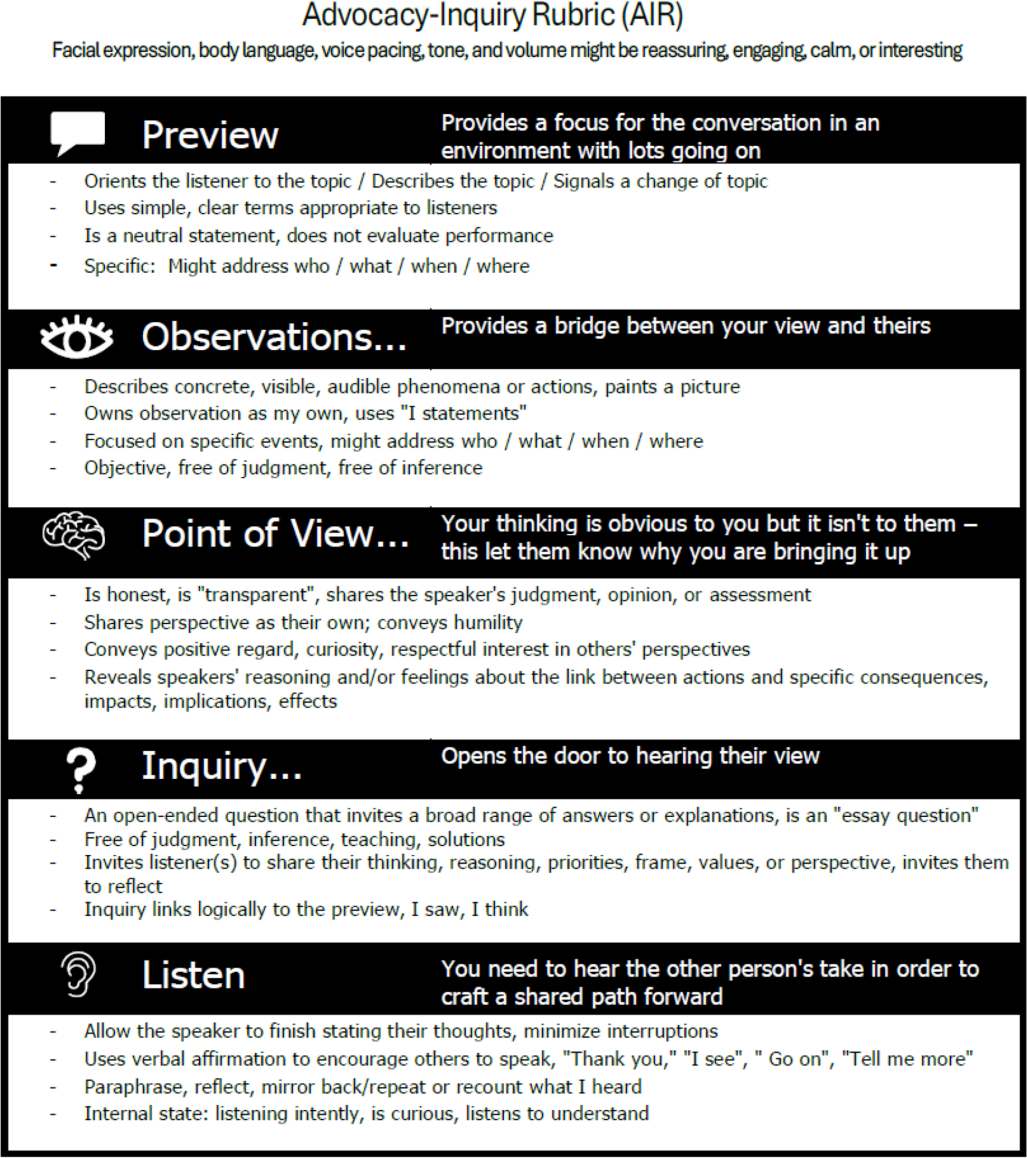
Teaching and Learning version of the Advocacy-Inquiry Rubric (AIR)

The *numeric AIR* is a 7-point BARS, ranging from “Ineffective” to “Extremely Effective” (Supplementary Material 3). The alignment of good and poor descriptors, as displayed in Supplementary Material 3, was developed in response to usability testing. The*teaching and learning AIR* provides four positive behavioral descriptors and no scale (Fig. 5). The *emoji AIR* is a 3-point BARS with sad, neutral, and smiling faces (Supplementary Material 4). In the numeric and emoji versions of the AIR, logically paired behavioral descriptors are linked with dotted lines, while unpaired behaviors are listed independently. A space for note-taking supports specific feedback. The numeric AIR aids formative or summative assessment and progress monitoring for simulation programs and their staff. The teaching and learning AIR supports those first learning debriefing and feedback and their facilitators. It introduces the Advocacy-Inquiry, and guides feedback.

The emoji AIR, invented in response to user interviews, supports peer assessment and feedback. We prioritized testing the emoji version and therefore have scoring inference validity evidence for the emoji scale, but not for the numeric scale. We collected ratings from three trained raters evaluating four elements in 22 Advocacy-Inquiry recordings for 88 individual ratings by each rater for each round, with additional ratings from the program creators for 648 total ratings. Our test of inter-rater reliability among three trained raters yielded good inter-rater consistency as measured by the intraclass correlation coefficient (ICC = 0.81) and good rater accuracy (ICC = 0.62). Raters trained to assess Advocacy-Inquiry using the rubric reported greater confidence and clarity about how to assess or provide feedback on the skills.

### Strengths and gaps in validity evidence for the AIR

Table 7 summarizes the strengths and gaps in validity evidence for the Advocacy-Inquiry Rubric (AIR) across the key inferences of scoring, generalization, extrapolation, and implication. This analysis also highlights the gaps requiring further research to solidify its validity evidence.

**Table 7:** Strengths and gaps in validity evidence for the AIR

Inference	AIR Development Step	Strengths and Gaps
**Scoring inference** An observation is reliably converted into a score or useful narrative for feedback.	Created a seven-point and three-point Behaviorally Anchored Rating Scale (BARS) with clear descriptors for good and poor performance of each element (Preview, Observation, Point of View, Inquiry, Listen) to guide in selecting, categorizing, and rating observed behaviors.	**Strengths:** Provided explicit scoring anchors that strengthen assessor’s ability to translate observation to score or narrative feedback. Strong empirical evidence of scoring inference for the emoji version of the scale: (ICC = 0.81) on novel, unscripted recordings of Advocacy-Inquiry statements from a one-day facilitator training course. **Gaps:** Tested scoring consistency in one context with one set of raters. No testing across diverse rater or facilitator populations or settings.
**Generalization inference** A specific score or qualitative assessment can be generalized to the other, related performance in the test “world.”	Delphi's process ensured that descriptors reflected an expert consensus on a range of good or poor behaviors.	**Strengths: **The range of behaviors captured in each descriptor counteracts overly narrow assessment. **Gaps**: Generalizability to other related skills (e.g. a full debriefing for feedback conversations, bedside teaching) or in different test contexts remains untested.
**Extrapolation inference** Whereas generalization takes us from a sample of observations of Advocacy-Inquiry in the test world to the universe of possible observations also in the test world, extrapolation evidence tells us whether measures in the test world can predict performance in the real world.	Relied on experts to ensure that descriptors reflected real-world Advocacy-Inquiry technique skills or behaviors.	**Strengths:** Strong alignment between rubric elements and real-world expert knowledge. **Gaps:** No direct evidence of how rubric performance relates to real-world impacts on full feedback, debriefing conversation skills, or learning outcomes.
**Implication inference(s)** In this step, we make a decision based on the assessment. For example, is the facilitator ready to assist or lead a learning conversation? Alternately, do facilitators feel more confident or better prepared as a result of getting feedback or an assessment based on the AIR?	Positioned the AIR as a formative and summative tool to improve Advocacy-Inquiry skills, debriefing quality, and feedback conversations in health professions education.	**Strengths:** Articulated clear intended uses and practical benefits of the rubric. Raters trained with the rubric reported greater clarity and confidence in assessing Advocacy-Inquiry skills. **Gaps:** Lacks longitudinal data to demonstrate real-world impacts on feedback, debriefing, or learning outcomes.
The “interpretation-use validity argument” [30] outlined here is a ladder of interlinked inferences that offer a window into the assumptions built into the assessment. To evaluate the validity, we look at evidence about the following links: (1) An observed performance can be linked to a single score; (2) this score links to performance in the test space; (3) performance in the test space extrapolates to real-world performance; and finally, (4) assessments of that performance relate to decisions about readiness in the real world.		

## Discussion

This study achieved four key outcomes: (1) definition of the construct of the Advocacy-Inquiry skill and intended uses of the AIR, (2) consensus-based development of behaviorally anchored descriptors for five established AI components via a Delphi method, (3) creation of three AIR versions tailored to different educational contexts, and (4) preliminary evidence of usability and inter-rater reliability for the numeric and emoji versions.

Advocacy-Inquiry has become a cornerstone conversational technique for simulation educators and leaders worldwide, offering a practical, transformative tool for feedback, debriefing, and navigating difficult conversations. Its broad acceptance lies in its ability to foster genuine, transparent sharing and nurture a spirit of curiosity and mutual discovery.

### Demystifying the Advocacy-Inquiry technique to achieve high-quality feedback and debriefing conversations

Vague standards regarding the complex skill of feedback and debriefing are often the norm, leaving facilitators and their training programs without a clear path to competence or mastery [46, 47]. To address this problem, we sought to demystify a core feedback and debriefing skill, Advocacy-Inquiry. We zoomed in on the behavioral markers that underlie the Preview, Observation, Point of View, Inquiry, and Listen microskills, making it easier for us to describe, learn, teach, and assess them.

### Promoting feedback and debriefing skills with the AIR

When we created the AIR, our goal was a rubric flexible enough to meet diverse skill-building needs. The result? Three formats. The *numeric AIR* (Supplementary Material 3) features good and poor performance descriptors on a seven-point scale, to be used for detailed assessment, tracking progress, and targeting improvements. We think it is best suited for mentors and programs with a developmental, non-punitive culture that supports both feedback and assessment. The *emoji AIR* (Supplementary Material 4) features the same good and poor performance descriptors but on a three-point emoji scale, to be used for peer-to-peer developmental assessment. We think it is best suited for programs in the early stages of building a peer-feedback culture. Finally, the *teaching and learning AIR* for novices in feedback and debriefing conversations and their facilitators (Fig. 5), simplifies things with only good performance descriptors. This format works well for faculty development in settings new to peer feedback and assessment or for those building foundational Advocacy-Inquiry skills. Users of this version are likely to experience lower cognitive load as they are not attending to the poor performance anchors. This applies both to raters and those performing the Advocacy-Inquiry technique.

We argue that the three formats share common strengths: they connect assessment of observable behaviors to feedback on feedback, and feedback on debriefing. They scaffold facilitators-in-training as they move from their native perspective to reliably interpreting behaviors through the lens of the rubric—what we call “seeing through the eyes of the rubric.”

### Lessons learned and next steps

We offer three lessons learned in the process of developing the AIR: the benefits of shared explicit standards, transparent validity arguments to enhance assessment, and the value of an international community of practice and the Delphi process.

#### The benefits of shared standards for accelerating learning

We envision the AIR joining the growing family of rubrics advancing communication skills in health professions education [48]. Building on the work of Buléon et al. and Schmutz et al., we believe the AIR, like other shared standards in health professions education, can accelerate competency development [27, 49] and is more robust because it was built through broad consensus [50]. We also think that rubrics are most effective when they support both formative feedback and summative assessment because they reinforce the pedagogical alignment between training and assessment, which legitimizes assessment following training [51], as the AIR does. Beyond setting targets, tools like the AIR help learners and coaches refine their mental models of effective communication, clarifying what success looks like.

#### Transparent validity argumentation as a model for fair and supportive assessment

As health professions education hurtles toward ever more competency-based standards of learning, the interpretation-use argument approach to validity offers a refreshing and transformative reminder to make our assumptions about assessment and feedback explicit (and not leave them tacit). This validity argument approach invites educators to agree on and explain the ladder of inferences built into the rubrics we use, and for what purposes the validity argument is made, e.g., developmental feedback versus summative feedback. This approach to “validating” instruments nudges us toward fairness and transparency.

Drawing on over 12 years of rater training experience, including work with the Debriefing Assessment for Simulation in Healthcare (DASH), we highlighted a critical but often overlooked step in building valid assessment: rater cognition. Moving from skill observation to scoring requires assessors to “see through the eyes of the instrument.” Without strengthening this link, the foundation of the validity argument is weak.

#### The Delphi process in an international community of practice

Developing a shared standard via four rounds of a Delphi technique may seem like a dry and pedantic exercise. However, we believe that 39 international expert panel members netted hidden benefits. It allowed them to come together across countries to articulate and share expertise each had built over years of debriefing and feedback practice. It allowed them to transform fragments of tacit knowledge into a coherent, explicit standard. It allowed space for both dominant and fringe ideas to be considered by experts making independent decisions. We think this collaborative effort was more than just standard-setting—it was knowledge production and a form of mutual learning and connection. The process highlighted the generative power of making implicit expertise explicit, strengthening both the rubric and the global community of practice.

#### Future applications of the Advocacy Inquiry Rubric

With its explicit behavioral markers and emphasis on supporting the tasks of rater cognition (selecting, categorizing, and then providing strong narrative judgments or rating scores), we believe the AIR can be used to create a new generation of rater training with two approaches. One is to create asynchronous rater training that strengthens rater cognition with built-in feedback on rating in iterative rounds of complexity. The other is to pre-train Artificial Intelligence programs (known as supervised machine learning) that allow instantaneous feedback on the facilitators-in-training’s ratings. Both of these approaches will reduce bottlenecks in rigorous competency or mastery assessment by preparing expert raters at scale [52].

### Limitations

The most important limitation of this effort is that the rubric could have been tested more extensively. It underwent usability testing, and the emoji version of the rubric was tested using a rater training process on one data set. While we have a high degree of confidence in the quality of the behavioral markers in the AIR and the level of consensus in the expert panel, how the instrument functions in assessments of feedback and debriefing conversations in diverse environments with a variety of raters is still to be determined. Finally, further work is needed to evaluate Advocacy-Inquiry skills as a proxy for overall feedback or debriefing skills, and to establish empirical cut scores or mastery levels for competency [53].

## Conclusion

The AIR represents an empirically derived behaviorally anchored rating scale to assess microskills of debriefing, namely Advocacy-Inquiry. A rigorous design process included debriefing experts from 12 countries and diverse practice settings, ensuring applicability across geographic and educational contexts. The rigorous and robust development of AIR lends itself to both research as well as a variety of formative and summative uses.

The impetus for creating the AIR was a desire to help facilitators refine these skills and enable simulation programs to elevate the quality of their learning conversations to meet the high standards now widespread in competency-based education. When we think of "rubrics" and "assessment," it is natural to focus on methodological or pedagogical concerns. Yet, at the heart of the AIR lies a more aspirational goal: improving communication across differences. By providing a shared framework, the AIR is not just an instrument for building skills but a bridge for fostering deeper understanding, respect, and curiosity. We hope it becomes a catalyst for thoughtful, adaptive learning through self-, peer-, and mentor-led feedback, helping us not just talk, but also connect.

## Supplementary Information

Supplementary Material 1: Supplemental Digital Content: Description of the questions for rounds 1, 2, 3, and 4 of the Delphi Method.

Supplementary Material 2.

Supplementary Material 3.

Supplementary Material 4.

## Data Availability

The data relating to the research is available in the article, in supplementary data, or upon reasonable request to the authors.
