# Supplementary material for: The Advocacy-Inquiry Rubric (AIR): a standard to build debriefing and feedback skills
Source: Adv Simul (Lond). 2025 Nov 24;10:60. doi: 10.1186/s41077-025-00381-z (PMC12645724; doi:10.1186/s41077-025-00381-z)
Supplement: Supplementary file 1 — Supplementary Material 1: Supplemental Digital Content: Description of the questions for rounds 1, 2, 3, and 4 of the Delphi Method. [file 41077_2025_381_MOESM1_ESM.docx]

**Supplemental Digital Content: Description of the questions for rounds 1, 2, 3, and 4 of the Delphi Method.**

| **Round 1** | |
| --- | --- |
| Question 1 | Define behavior(s) that comprise a good [ELEMENT NAME]. Give example(s) if you can.  Define behavior(s) that comprise a poor [ELEMENT NAME]. Give example(s) if you can. |
| **Round 2** | |
| Question 1 | Rate the importance of each descriptor below. You are asked to rate these in terms of how important you think they are in teaching or learning a GOOD [ELEMENT NAME].  Rate the importance of each descriptor below. You are asked to rate these in terms of how important you think they are to contributing to a POOR [ELEMENT NAME].  *Likert importance scale^a^* |
| **Round 3** | |
| Question 1 | These 3 descriptors were rated relatively high in their importance in teaching and learning this skill. Do you agree they should be kept in the final list for a GOOD/POOR [ELEMENT NAME]?  *Binary: Yes/No per each descriptor.* |
| Question 2 | These 4 descriptors were rated a bit lower in their importance in teaching and learning this skill. Use your mouse to order them in terms of their importance for a GOOD/POOR [ELEMENT NAME] (most important on top).  *Descriptors appeared in a random order at each time.* |
| Question 3 | These descriptors were rated lowest in their importance in teaching and learning this skill. Do you agree they should be discarded from the final list for a GOOD/POOR [ELEMENT NAME]?  *Binary: Yes/No per each descriptor.* |
| **Round 4** | |
| Question 1 | These 6 descriptors have been selected through the Delphi Method to characterize a GOOD/POOR [ELEMENT NAME]. Do you agree they should be the final list for a GOOD/POOR [ELEMENT NAME]?  *Binary: Yes/No per each descriptor.* |
| Question 2 | Please rate each descriptor for a GOOD/POOR [ELEMENT NAME] with what you expect of a beginner, intermediate, or advanced practitioner of the method.  *Rating as beginner, intermediate, or advanced without a predefined number of descriptors per category.* |
| ^a^Likert importance scale (6 grades): Extremely important, Very important, Important, Somewhat important, Neither important or unimportant, Unimportant.  For each Round, an open box allows optional comments. | |
