# Supplementary material for: The Advocacy-Inquiry Rubric (AIR): a standard to build debriefing and feedback skills
Source: Adv Simul (Lond). 2025 Nov 24;10:60. doi: 10.1186/s41077-025-00381-z (PMC12645724; doi:10.1186/s41077-025-00381-z)
Supplement: Supplementary file 2 — Supplementary Material 2. [file 41077_2025_381_MOESM2_ESM.pdf]

Professions	Number (%)
Advance practice nurse	3 (8)
Educator/Researcher	11 (28)
Nurse	4 (10)
Physician	18 (46)
No Response	3 (8)

Advanced Degrees	Number (%)
Bachelor of Sciences	4 (10)
Master in Education	9 (23)
Master of Sciences	8 (21)
Medical Doctor	18 (46)
Academic Doctorate (PhD; EdD)	16 (41)
Registered Nurse	8 (21)

Experts are from 13 countries: Australia (4), Belgium (1), Colombia (1), France (1), Germany (2), Hong Kong (2), Mexico (1), New Zealand (1), Saudi Arabia (2), Spain (2), Switzerland (1), United Kingdom (1) and United States of America (20). Two did not answer.

Questions	Median (min – max)
How many years have you been **involved with simulation**? (year)	11.5 (5 – 25)
Estimate how many **debriefing** or formal feedback sessions within a course or clinical setting you have conducted in the last two years. (number)	78 (8 – 500)
Approximately, How many years have you been **teaching others how to debrief** or to give formal feedback? (year)	8.5 (2 – 25)
Estimate how many **debriefing the debriefing or feedback on feedback** (in course, informal or in workshop) you have conducted in the last two years? (number)	40 (5 – 200)
Approximately, how many **research studies** not yet published on the topic of debriefing, feedback, debriefing of the debriefing or feedback on feedback have you been involved in any way? (number)	2 (0 – 10)
Approximately, how many **abstracts** not yet published on the topic of debriefing, feedback, debriefing of the debriefing or feedback on feedback, have you been involved in any way? (number)	1.5 (0 – 30)
Approximately how many peer-reviewed publications not yet published on the topic of debriefing, feedback, debriefing of the debriefing, or feedback on feedback, have you been involved in any way? (number)	1 (0 – 25)

	Pre-Licensure students	Advanced Training e.g. residents	Practicing Clinicians
Interprofessional teams	10	25	34
Nurses	7	11	30
Paramedics	0	1	12
Physicians	14	23	33
Therapists (Physio, Occupational, Respiratory)	3	4	20
Others	5	7	18

Experts have taught the debriefing feedback molecule in 29 countries: Argentina (4), Australia (12), Austria (1), Belgium (4), Brazil (3), Canada (8), Chile (5), China (2), Colombia (5), Costa Rica (1), Ecuador (1), France (4), Germany (7), Hong Kong (10), Italy (1), Mexico (6), New Zealand (4), Peru (2), Portugal (1), Qatar (2), Saudi Arabia (6), Slovakia (1), Singapore (6), Spain (9), Switzerland (2), United Arab Emirates (5), United Kingdom (5), United States of America (25), and Uruguay (1).

Experts have taught the debriefing feedback molecule in 5 languages: Cantonese (3), English (35), French (2), German (3), and Spanish (8).
