# Supplementary material for: The Advocacy-Inquiry Rubric (AIR): a standard to build debriefing and feedback skills
Source: Adv Simul (Lond). 2025 Nov 24;10:60. doi: 10.1186/s41077-025-00381-z (PMC12645724; doi:10.1186/s41077-025-00381-z)
Supplement: Supplementary file 3 — Supplementary Material 3. [file 41077_2025_381_MOESM3_ESM.pdf]

Advocacy-Inquiry Rubric

Rating Preview, I Saw, I Think, I Wonder, Listen

This process helps you view conversational skills as an expert would, in order to help you and your peers develop skills as expert communicators.

You will reflect on and rate the elements of an observed Advocacy-Inquiry.

Element “Preview”

Ineffective		Effective
	Notes:	
Off-putting words, threatening language		Orients the listener to the topic/Describes the topic/Signals a change of topic
No preview or signal of topic change		Uses simple, clear terms appropriate to listeners
Points out or "calls out" individuals in an unwelcome way		Is a neutral statement, does not evaluate performance
Misleading preview		Specific: Might address who/what/when/where
Includes assumptions or inferences		Seeks permission/Invites to discuss
Includes a judgment (may be hidden), or an assessment of performance		Is succinct, as concise as possible

Rating Scale (Ratings are holistic, not arithmetic)

1	2	3	4	5	6	7
Ineffective	Somewhat Ineffective	Marginally Effective	Somewhat Effective	Mostly Effective	Very Effective	Extremely Effective

Rate the elements of the Advocacy Inquiry using the effectiveness scale. Think holistically and not arithmetically as you consider the cumulative impact of behaviors, which may not bear equal weight. Some behaviors vary by degree; some are present, and some are not. You must weigh the impact of variation in the behaviors as you see fit based on your holistic view of the element.

Element "I Saw/I Heard" (Observation)

Ineffective		Effective
	Notes:	
Does not include an observation, no "I saw/I heard" statement		Describes concrete, visible, audible phenomena or actions, paints a picture
Verbal statements are accusatory; may appear to blame a person or persons		Owns observation as my own, uses "I statements"
Vague, too general, too abstract, does not refer to observable phenomena		Focused on specific events, might address who/what/when/where
Includes judgment, critique		Objective, free of judgment, free of inference
Presents observations as "the truth", as certain, does not "own" the observation as the speaker's perspective		Connects to the preview and upcoming "I think"
Includes inferences or assumptions about others, ascribes motives, feelings, or thoughts		Reveals the speaker's areas of uncertainty (e.g. what they didn't hear or see clearly)

Rating Scale (Ratings are holistic, not arithmetic)

**1**	**2**	**3**	**4**	**5**	**6**	**7**
Ineffective	Somewhat Ineffective	Marginally Effective	Somewhat Effective	Mostly Effective	Very Effective	Extremely Effective

Rate the elements of the Advocacy Inquiry using the effectiveness scale. Think holistically and not arithmetically as you consider the cumulative impact of behaviors, which may not bear equal weight. Some behaviors vary by degree; some are present, and some are not. You must weigh the impact of variation in the behaviors as you see fit based on your holistic view of the element.

Element “I Think” (Point of View)

Ineffective		Effective
	Notes:	
Verbal statements are accusatory or aggressive; appear to blame or humiliate a person or persons		Is honest, is "transparent", shares the speaker's judgment, opinion, or assessment
Includes condemnation of a person or team, mistakes spotlighted as a violation		Shares perspective as their own; conveys humility
Speaker omits statements of their point of view completely		Conveys positive regard, curiosity, respectful interest in others' perspectives
The speaker's reasoning, judgment, opinion, or take on the link between actions and results is missing, implied, cloaked, sugar-coated, or too vague		Reveals speakers' reasoning and/or feelings about the link between actions and specific consequences, impacts, implications, effects
Presents own perspective as "the truth", conveys certainty, appears to close off other perspectives		Connects to the preview, "I saw" in a powerful way
Includes inferences or assumptions about others, ascribes motives, feelings, or thoughts		Normalizes the performance (if appropriate)

Rating Scale (Ratings are holistic, not arithmetic)

1	2	3	4	5	6	7
Ineffective	Somewhat Ineffective	Marginally Effective	Somewhat Effective	Mostly Effective	Very Effective	Extremely Effective

Rate the elements of the Advocacy Inquiry using the effectiveness scale. Think holistically and not arithmetically as you consider the cumulative impact of behaviors, which may not bear equal weight. Some behaviors vary by degree; some are present, and some are not. You must weigh the impact of variation in the behaviors as you see fit based on your holistic view of the element.

Element “I Wonder” (Inquiry)

Ineffective		Effective
	Notes:	
Closed-ended, leading, or yes/no question, may start with did/didn't, would/wouldn't, is/isn't, don't you think		An open-ended question that invites a broad range of answers or explanations, is an "essay question"
Conveys judgment, condemnation, is an inquisition rather than an inquiry		Free of judgment, inference, teaching, solutions
Is a "test" question to assess knowledge (without a preview about the reason for the question)		Invites listener(s) to share their thinking, reasoning, priorities, frame, values, or perspective, invites them to reflect
"Guess what I am thinking" question, appears to explore thinking but seeks an answer the speaker has in mind already		Inquiry links logically to the preview, I saw, I think
Includes inferences or assumptions in the question, ascribes motives, feelings, or thoughts		Is short, is concise as possible
Conveys certainty, lacks curiosity		Conveys genuine curiosity, interest, wonder

Rating Scale (Ratings are holistic, not arithmetic)

**1**	**2**	**3**	**4**	**5**	**6**	**7**
Ineffective	Somewhat Ineffective	Marginally Effective	Somewhat Effective	Mostly Effective	Very Effective	Extremely Effective

Rate the elements of the Advocacy Inquiry using the effectiveness scale. Think holistically and not arithmetically as you consider the cumulative impact of behaviors, which may not bear equal weight. Some behaviors vary by degree; some are present, and some are not. You must weigh the impact of variation in the behaviors as you see fit based on your holistic view of the element.

Element "Listen"

Ineffective	Effective
Notes:	
Interrupts, talks over, cuts people off too often	Allow the speaker to finish stating their thoughts, minimize interruptions
Voice tone, words, or paravocal sounds (sighing, sniffing, grunting, harsh laughter, tongue clicking, muttering under one's breath) convey disdain, condemnation, anger, suspicion	Uses verbal affirmation to encourage others to speak, "Thank you," "I see", " Go on", "Tell me more"
Arguing in a way that suppresses other person's sharing their point of view	Paraphrase, reflect, mirror back/repeat or recount what I heard
Lecturing or talking ad nauseam	Internal state: listening intently, is curious, listens to understand
Dismissing other person's worries, concerns, focus	Clarifies or tests own understanding: invites clarification, expansion, deeper explanation
Correcting or interpreting other people's thoughts in a way that suppresses their talking	Allows silence

Rating Scale (Ratings are holistic, not arithmetic)

**1**	**2**	**3**	**4**	**5**	**6**	**7**
Ineffective	Somewhat Ineffective	Marginally Effective	Somewhat Effective	Mostly Effective	Very Effective	Extremely Effective

Rate the elements of the Advocacy Inquiry using the effectiveness scale. Think holistically and not arithmetically as you consider the cumulative impact of behaviors, which may not bear equal weight. Some behaviors vary by degree; some are present, and some are not. You must weigh the impact of variation in the behaviors as you see fit based on your holistic view of the element.
