# Supplementary material for: The Advocacy-Inquiry Rubric (AIR): a standard to build debriefing and feedback skills
Source: Adv Simul (Lond). 2025 Nov 24;10:60. doi: 10.1186/s41077-025-00381-z (PMC12645724; doi:10.1186/s41077-025-00381-z)
Supplement: Supplementary file 4 — Supplementary Material 4. [file 41077_2025_381_MOESM4_ESM.pdf]

Advocacy-Inquiry Rubric

Rating Preview, I Saw, I Think, I Wonder, Listen

This process helps you view conversational skills to assist you and your peers in developing skills as expert communicators.

You will reflect on and rate the elements of an observed Advocacy-Inquiry.

Element "Preview"

Ineffective		Effective
	Notes:	
Off-putting words, threatening language		
No preview or signal of topic change	<------------------------------>	Orients the listener to the topic/Describes the topic/Signals a change of topic
		Uses simple, clear terms appropriate to listeners
Misleading preview		
Points out or "calls out" individuals in an unwelcome way	<------------------------------>	Specific: Might address who/what/when/where
Includes a judgment (may be hidden), or an assessment of performance	<------------------------------>	Is a neutral statement, does not evaluate performance
Includes assumptions or inferences		
		Seeks permission/Invites to discuss
		Is succinct, as concise as possible

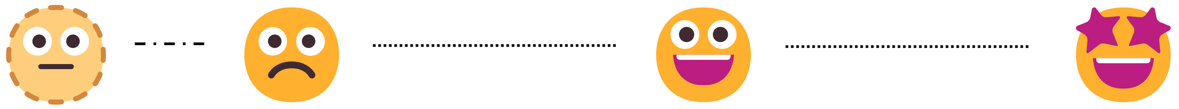

Not Observed Ineffective Somewhat Effective Extremely Effective

Rate the elements of the Advocacy Inquiry using the emoji scale. Think holistically as you consider the cumulative impact of behaviors which may not bear equal weight. Some behaviors vary by degree, some are present or not present. Based on your holistic view of the element, you must weigh the impact of variation in the behaviors as you see fit.

Element “I Saw/I Heard” (Observation)

Ineffective	*Notes:*	Effective
Verbal statements are accusatory; may appear to blame a person or persons		
Vague, too general, too abstract, does not refer to observable phenomena	←-----------------------------→	Describes concrete, visible, audible phenomena or actions, paints a picture
Does not include an observation, no "I saw/I heard" statement	←-----------------------------→	Owns observation as my own, uses "I statements"
		Focused on specific events, might address who/what/when/where
Includes judgment, critique	←-----------------------------→	Objective, free of judgment, free of inference
Presents observations as "the truth", as certain, does not "own" the observation as the speaker's perspective		
Includes inferences or assumptions about others, ascribes motives, feelings, or thoughts	←-----------------------------→	Objective, free of judgment, free of inference
		Connects to the preview and upcoming "I think"
		Reveals the speaker's areas of uncertainty (e.g. what they didn't hear or see clearly)

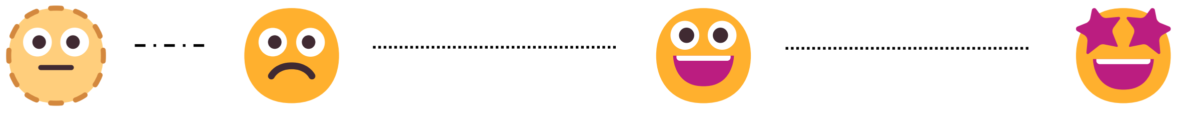

Not Observed — Ineffective — Somewhat Effective — Extremely Effective

Rate the elements of the Advocacy Inquiry using the emoji scale. Think holistically as you consider the cumulative impact of behaviors which may not bear equal weight. Some behaviors vary by degree, some are present or not present. Based on your holistic view of the element, you must weigh the impact of variation in the behaviors as you see fit.

Element “I Think” (Point of View)

Ineffective	*Notes:*	Effective
Verbal statements are accusatory or aggressive; appear to blame or humiliate a person or persons		
Includes condemnation of a person or team, mistakes spotlighted as a violation		
Speaker omits statements of their point of view completely	↔	Is honest, is "transparent", shares the speaker's judgment, opinion, or assessment
Presents own perspective as "the truth", conveys certainty, appears to close off other perspectives	↔	Shares perspective as their own; conveys humility
		Conveys positive regard, curiosity, respectful interest in others' perspectives
The speaker's reasoning, judgment, opinion, or take on the link between actions and results is missing, implied, cloaked, sugar-coated, or too vague	↔	Reveals speakers' reasoning and/or feelings about the link between actions and specific consequences, impacts, implications, effects
		Connects to the preview, "I saw" in a powerful way
Includes inferences or assumptions about others, ascribes motives, feelings, or thoughts		
		Normalizes the performance (if appropriate)

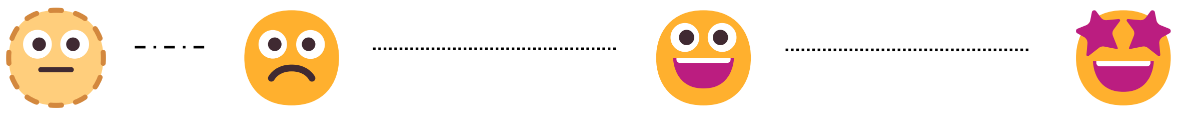

Not Observed — Ineffective — Somewhat Effective — Extremely Effective

Rate the elements of the Advocacy Inquiry using the emoji scale. Think holistically as you consider the cumulative impact of behaviors which may not bear equal weight. Some behaviors vary by degree, some are present or not present. Based on your holistic view of the element, you must weigh the impact of variation in the behaviors as you see fit.

Element "I Wonder" (Inquiry)

Ineffective		Effective
	Notes:	
Closed-ended, leading, or yes/no question, may start with did/didn't, would/wouldn't, is/isn't, don't you think	<----------------------------->	An open-ended question that invites a broad range of answers or explanations, is an "essay question"
Conveys judgment, condemnation, is an inquisition rather than an inquiry	<----------------------------->	Free of judgment, inference, teaching, solutions
Is a "test" question to assess knowledge (without a preview about the reason for the question)		
		Invites listener(s) to share their thinking, reasoning, priorities, frame, values, or perspective, invites them to reflect
		Inquiry links logically to the preview, I saw, I think
Conveys certainty, lacks curiosity	<----------------------------->	Conveys genuine curiosity, interest, wonder
"Guess what I am thinking" question, appears to explore thinking but seeks an answer the speaker has in mind already		
Includes inferences or assumptions in the question, ascribes motives, feelings, or thoughts		
		Is short, is concise as possible

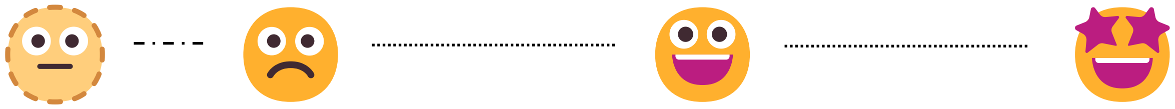

Not Observed   Ineffective   Somewhat Effective   Extremely Effective

Rate the elements of the Advocacy Inquiry using the emoji scale. Think holistically as you consider the cumulative impact of behaviors which may not bear equal weight. Some behaviors vary by degree, some are present or not present. Based on your holistic view of the element, you must weigh the impact of variation in the behaviors as you see fit.

Element "Listen"

Ineffective		Effective
	Notes:	
Interrupts, talks over, cuts people off too often	<------------------->	Allow the speaker to finish stating their thoughts, minimize interruptions
Voice tone, words, or paravocal sounds (sighing, sniffing, grunting, harsh laughter, tongue clicking, muttering under one's breath) convey disdain, condemnation, anger, suspicion		
		Uses verbal affirmation to encourage others to speak, "Thank you," "I see", " Go on", "Tell me more"
Arguing in a way that suppresses other person's sharing their point of view		
Lecturing or talking ad nauseam		
		Paraphrase, reflect, mirror back/repeat or recount what I heard
		Internal state: listening intently, is curious, listens to understand
Dismissing other person's worries, concerns, focus		
		Allows silence
Correcting or interpreting other people's thoughts in a way that suppresses their talking	<------------------->	Clarifies or test own understanding: invites clarification, expansion, deeper explanation

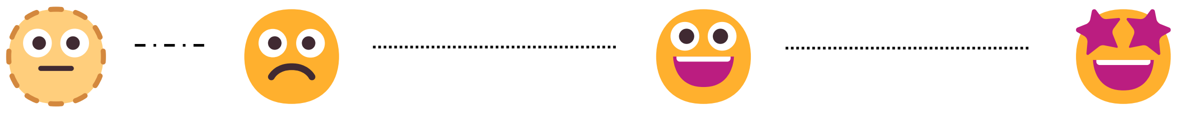

Not Observed   Ineffective   Somewhat Effective   Extremely Effective

Rate the elements of the Advocacy Inquiry using the emoji scale. Think holistically as you consider the cumulative impact of behaviors which may not bear equal weight. Some behaviors vary by degree, some are present or not present. Based on your holistic view of the element, you must weigh the impact of variation in the behaviors as you see fit.
